# Hamilton on Nervous Diseases

**Published:** 1878-12

**Authors:** 


### Hamilton on Nervous Diseases.

Efforts having been made to prejudice the profession against Dr. Hamilton's recent work on Nervous Diseases, attention is called to the following estimate of the volume, from the October number of "Brain," the leading neurological journal of England. The review is signed by J. Crichton Browne, who is well known as one of the ablest of English neurologists.—

Dr. McLane Hamilton may be congratulated on having produced a concise and practical book on nervous diseases, and on having thus achieved the object which he prescribed to himself on commencing the laborious literary task, the fruits of which now lie before us in a large, well-arranged and well-printed volume. This is, unquestionably, the best and most complete text-book of nervous diseases that has yet appeared, and were international jealousy in scientific affairs at all possible, we might be excused for a feeling of chagrin that it should be of American parentage. A glance at its pages shows that the greater bulk of the material of which it is composed has been derived from English sources, and it might naturally have been anticipated that the digestion and compilation of that material would be accomplished in England. This work, however, has been performed in New York, and has been so well performed that no room is left for anything but commendation. With great skill Dr. Hamilton has presented to his readers a succinct and lucid survey of all that is known of the pathology of the nervous system, viewed in the light of the most recent researches. In so comprehensive an undertaking, involving such a number of moot questions, it is of course inevitable that criticism should be challenged on many pages. Frequently we find ourselves differing from Dr. Hamilton's conclusions and recommendations; but to discuss the points of divergence would be to enter upon the details, and leave untouched the general scope of the volume. From the preliminary description of the methods of examination and study, and of the instruments of precision employed in the investigation of nervous diseases, up till the final collection of formulæ, the book is eminently practical. It brings together much scattered information in a methodical and well-balanced manner We think, however, that it would enhance its usefulness if, in future editions, Dr. Hamilton would give more prominence than he has now done to the physical symptoms of, nervous diseases; if he would supply the specific indications for treatments and fuller directions for the employment of his formulæ, in the manner of Tanner's 'Practice of Medicine'; and if he would amend his pictorial illustrations, which as they now stand are the weakest part of his work, having neither artistic nor diagrammatic merit."

